# GABA-Aα5 Might Be Involved in Learning-Memory Dysfunction in the Offsprings of Chronic Ethanol-Treated Rats via GABA-Aα5 Histone H3K9 Acetylation

**DOI:** 10.3389/fnins.2019.01076

**Published:** 2019-10-16

**Authors:** Kuan Zeng, Aimin Xie, Xiaojie Dong, Jia Jiang, Wei Hao, Min Jiang, Xuebing Liu

**Affiliations:** ^1^Wuhan Mental Health Center, Wuhan, China; ^2^Applied Psychology, Marx College, China University of Geosciences, Wuhan, China; ^3^Key Laboratory of Psychiatry and Mental Health of Hunan Province, National Clinical Research Center for Mental Disorders, Mental Health Institute of the Second Xiangya Hospital, National Technology Institute of Psychiatry, Central South University, Changsha, China

**Keywords:** ethanol, addiction, GABA-Aα5, histone H3K9 acetylation, offspring

## Abstract

Recently, numerous studies have been focused on the relationship between GABA-A receptors and alcohol-induced spatial learning and memory deficits. GABA-Aα5, a subunit of GABA-A receptors, is considered to play an important role in alcohol-induced cognitive impairment, however, the mechanism remains obscure. In this study, we found that the expression of GABA-Aα5 increased in rats treated with chronic ethanol via histone H3K9 acetylation. Furthermore, this epigenetic modification could be inherited by the next generations, which eventually exhibit similar spatial learning and memory deficits in the offsprings. In summary, our results suggested that GABA-Aα5 might be involved in chronic ethanol treatment-induced learning-memory dysfunction and for the first time proved that learning-memory dysfunction could be inherited by the offsprings via histone H3K9 acetylation. Hopefully, in the near future, GABA-Aα5 inhibitors would be an effective way to treat alcohol-induced cognition impairment.

## Introduction

More than three million people died of alcohol abuse in 2016, as reported by the World Health Organization (WHO) in 2018 (World Health Organization [WHO], 2018). Alcohol abuse has been considered as the trigger of more than 5% of the global disease burden. According to the record, 3.3 million people died of alcohol in 2012, accounting for up to 5.9% of all deaths worldwide reported by the WHO. Besides, a recent study also showed that the prevalence of age-standardized alcohol dependence was 843.2 per 100,000 people in 2015 (Peacock et al., 2018). Herein, a cure against alcohol-induced damage is an emerging demand.

Numerous researches have been focused on alcohol metabolism and addiction (Berkel and Pandey, 2017; Pandey et al., 2017; Tulisiak et al., 2017) and gamma-aminobutyric acid (GABA), one of the major inhibitory neurotransmitters in the central nervous system. It is widely acknowledged that GABA receptors play an important role in multiple neurobehavioral responses to alcohol, especially GABA-A receptors (Tiwari et al., 2014; Olsen and Liang, 2017; Azevedo and Mammis, 2018; Rossi and Richardson, 2018). GABA-A receptors were considered to be associated with alcohol tolerance, dependence, and withdrawal (Enoch, 2008). Collinson et al. (2002) proposed that both learning and memory were enhanced in mice lacking the α5 subunit of the GABA-A receptor. Chester and Cunningham (2002) demonstrated that GABA-Aα5 inhibitors alleviated the alcohol-induced memory loss. Moreover, Alpha5IA, an inverse agonist selective for GABA-Aα5, could enhance cognition (Dawson et al., 2006). It is concluded that GABA-Aα5 is of significant importance in alcohol-induced cognitive impairment, however, the mechanism remains obscure.

To gain further insights on alcohol-induced cognition impairment and GABA-Aα5, we used chronic ethanol treatment rats to investigate the potential association in between. Furthermore, this study was also designed to explore whether the learning-memory dysfunction could be inherited by the offsprings.

## Materials and Methods

### Materials

Anhydrous ethanol was purchased from Tianjin De’en Chemical Reagents Co., Ltd. (Tianjin, China). Sodium pentobarbital was purchased from Sigma (United States). Antibody was obtained from Sigma (United States). All chemicals were of the highest level available.

### Animal Experiments

All adult Sprague–Dawley (SD) rats were provided by the Henan Animal Experimental Center and group housed with free access to water and food in established animal houses in thermoregulated environment (25 ± 0.5°C and 55% humidity) with a 12-h light/dark cycle. Rats were picked up to adapt to the environment 1 week before the experiments. In the first 3 days of the experiment, the operator touched and grasped the rats briefly (3 min/rat/day) to promote adaptation. All experiments were conducted in accordance with the animal experiment regulations of Xiang Ya Medical School and Xin Xiang Medical College of Central South University, and efforts were made to minimize suffering.

All 24 adult rats weighed 190–225 g, including 12 females and 12 males, and were randomly divided into four groups by gender (six rats for each gender). At 2 months of age, pure saline and ethanol-containing saline were prepared for chronic ethanol training. In the training period, the amount of 0.5 g/kg of saline or 5 g/kg of ethanol was administered by intraperitoneal injection for 15 days. As a result, four groups were obtained, as follows: male group treated with saline (MS), female group treated with saline (FS), male group treated with ethanol (ME), and female group treated with ethanol (FE). Ethanol-treated rats were used to perform Morris water maze (MWM) tests, followed with quantitative real-time polymerase chain reaction (RT-PCR) and chromatin immunoprecipitation (ChIP).

To obtain offsprings, trained rats were paired as ME + FE, ME + FS, FE + MS, and FS + MS. All offsprings were grouped by their parents as ME + FE (MEFE), ME + FS (MEFS), FE + MS (FEMS), and FS + MS (FSMS) and were sacrificed to obtain brain tissue at 3 months of age.

### Morris Water Maze Tests

Morris Water Maze testing was conducted in a round stainless steel water tank with 200 cm in diameter and 50 cm in height and filled to a depth of 30 cm with water at 22–26°C; a submerged transparent platform was placed 30 cm from the pool’s edge and submerged 2 cm beneath the water surface. The platform remained in the same position throughout the trials.

All the rats were trained with four trials per day at intervals of 60 s from different quadrants of the tank for 4 days. The starting point was changed for each trial. Rats were required to swim until they reached the platform. If the rat failed to reach the platform within 60 s, it was gently guided to the platform and allowed to rest for 30 s. In the fifth day, each animal was tested in four, single 60 s sessions from different quadrants. The escape latency was recorded using an automatic photographic recording and analysis system (Smart Systems Technology Corporation Ltd., Shanghai, China). If the rat failed to reach the platform, testing score was recorded for 60 s. The mean time of the four escape latencies would be the value of the whole day.

### Quantitative Real-Time Polymerase Chain Reaction

Rats were sacrificed to harvest hippocampus tissue at 3 months of age. Total RNA from the hippocampus was extracted using TRIzol Reagent (Invitrogen, Waltham, MA, United States). First-strand complementary DNA (cDNA) was synthesized from 500-ng total RNA using a high-capacity cDNA reverse transcription kit (Applied Invitrogen, Shanghai). PCR was performed on an Applied Biosystems 7900 Prism Real-Time PCR system and SYBR Premix Ex Taq (TaKaRa, Dalian, Japan) in a standard PCR mixture of 10 μl prepared in duplicate according to the manufacturer’s protocol. Quantitative PCR primers for GABA-Aα5 were as follows: 5′-AAGATGAAAGGCTGCGGTTTA-3′ and 5′-CCATGAGGTGGTACTGGTTGA-3′. PCR primers of GAPDH were 5′-CGTAGCTCAGGCCTCTGCGCCCTT-3′ and 5′-CTGGCACTGCACAAGAAGATGCGGCTG-3′. The size of the PCR product is 372 bp, and the PCR cycling conditions were 95°C (5 min), 95°C (30 s), 60°C (45 s), 72°C (45 s), and 72°C (5 min, 35 cycles). Dissociation curve was obtained after RT-PCR, and all the dissociation curves showed a single peak, which suggested that the reaction has high specificity. The cDNA amplification reaction was performed in the presence of SYBR Green in triplicate. The CT value of each sample was obtained using FTC-2000 software. To normalize each sample, GAPDH, the normal housekeeping gene, was used as an internal reference. By using the delta-delta CT method, multiple changes in the mRNA levels and control value were calculated to compare the relative expression results among different treatments. The relative expression of mRNA in the experimental group compared with the control group was calculated using the 2-ΔΔCT method. ΔCT = CT (GABA-Aα5)-CT (GAPDH), ΔΔCT = ΔCT (experimental group)-ΔCT (control group).

### Chromatin Immunoprecipitation

This procedure was performed based on the instruction of the ChIP kit (number 17-371, Millipore) with a few modifications. In brief, frozen prefrontal cortex (PFC) tissue was cross-linked in formaldehyde for 10 min and then terminated by glycine. Chromatin was then extracted using sodium dodecyl sulfate (SDS) lysis buffer, followed with optimal sonication to harvest 400–500 bp of fragments. The diluted samples were further grouped into the positive control (anti-RNA Polymerase II), negative control (normal mouse IgG), and anti-acetylation. Salmon sperm DNA/protein G agarose was used to preclear all the samples. After a short centrifugation, the supernatant was harvested for immunoprecipitation with antibodies directed against H3 acetylation on Lys9 (kit number 9671, Cell Signaling Technology), tri-methyl-H3 (Lys4) (kit number 9727, Cell Signaling Technology), RNA polymerase II, and normal mouse IgG overnight at 4°C. Salmon sperm DNA/protein G agarose beads were collected and washed with elution buffer to obtain targeted DNA–histone complexes. All immunoprecipitates were incubated at 65°C for 5 h and further incubated with RNase A at 37°C for 30 min. Proteinase K was used to purify the DNA associated with acetylated histones. Most ChIP experiments were performed twice on two independent tissue samples for confirmation.

### Statistical Analysis

A two-sample *t*-test was used for comparison between different groups. The least significant difference (LSD) method was used on the condition that the variance was homogeneous; if the variance was unequal between groups, the Dunnett T3 method was used. All data analyses were performed on SPSS 19.0 and presented as mean ± SD.

## Results

### Chronic Ethanol Treatment-Induced Spatial Learning and Memory Deficits

**FIGURE 1:**
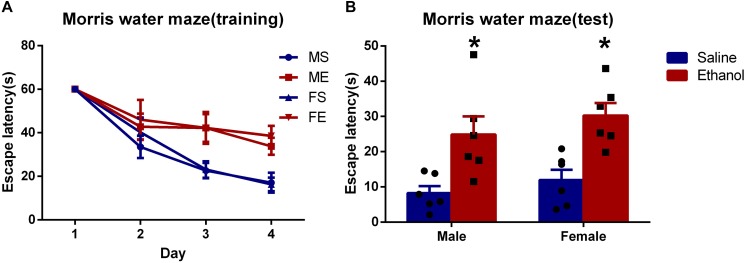
Chronic ethanol treatment-induced spatial learning and memory deficits. **(A)** The average escape latency during in training period. **(B)** The average escape latency on the fifth day. All data are presented as mean ± SD (*n* = 6). ^∗^*P* < 0.05 compared with the control group (treated with saline).

To explore the effect on spatial learning and memory after chronic ethanol treatment, MWM tests were performed. As suggested in Figure 1, no significance difference was seen in the escape latency in the first 2 days among the four groups. However, in the following two training days (day 3 and day 4), chronic ethanol-treated groups (ME and FE) took significantly longer time to reach the platform than did the rats treated with saline, MS and FS (*P* < 0.05 each day). In day 5, the escape latency of ethanol-treated groups was significantly longer than that of saline-treated control groups (MS vs. ME, and FS vs. FE, Figure 1). The results suggested that the chronic ethanol treatment had induced spatial learning and memory deficits.

### Chronic Ethanol Treatment Increased GABA-Aα5 Expression at the mRNA Level

**FIGURE 2:**
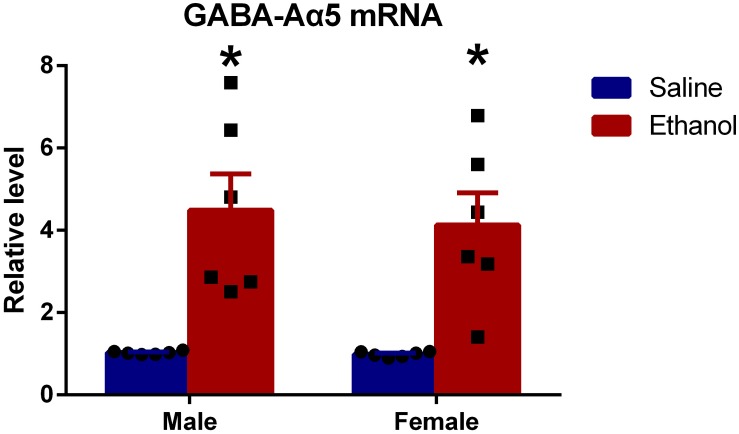
Chronic ethanol treatment increased GABA-Aα5 expression at the mRNA level. All data are presented as mean ± SD (*n* = 6). ^∗^*P* < 0.05 compared with the control group.

To further investigate whether GABA-Aα5 was involved in chronic ethanol-induced impairments, we measured the mRNA expression of GABA-Aα5. GAPDH was used as an internal control for normalization. As shown in Figure 2, compared with MS, the mRNA expression of ME was significantly higher (*P* < 0.05). A similar result was also found in FS and FE groups, which indicated that GABA-A mRNA expression was elevated after chronic ethanol treatment.

### Increased GABA-Aα5 Histone H3K9 Acetylation Might Be the Cause of GABA-Aα5 Expression Upregulation

It is widely acknowledged that epigenetic regulation, including DNA methylation, histone methylation, and acetylation, plays a great role in protein expression (Tsankova et al., 2007; Mitchelmore and Gede, 2014; Dogra et al., 2016). Particularly histone modifications, as one of the major epigenetic mechanisms, are extensively studied (Kuehner et al., 2019). Robinson et al. (2012) showed that the acetylation of histone H3K9 in the promoter region GABA-Aα5 gene was associated with its expression. To gain detailed information, we measured histone H3K9 acetylation in GABA-Aα5 gene by ChIP.

**FIGURE 3:**
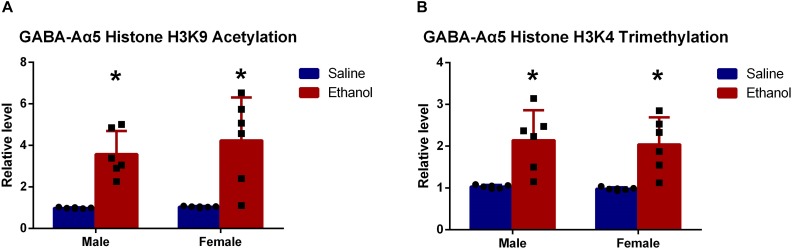
GABA-Aα5 histone H3K9 acetylation **(A)** and trimethylation level **(B)** were upregulated in chronic ethanol-treated groups. All data are presented as mean ± SD (*n* = 6). ^∗^*P* < 0.05 compared with the control group.

Results showed that histone H3K9 acetylation level in chronic ethanol-treated groups (both female and male rats) was significantly higher than that of saline-treated groups (Figure 3A, *P* < 0.05). This indicated that histone H3K9 acetylation might be responsible for the increased GABA-Aα5 mRNA level.

As demonstrated by our previous work, the upregulation of histone H3K4 trimethylation also led to increased GABA-Aα5 expression (Zeng et al., 2018), histone H3K9 trimethylation level was also examined. As expected, it was also found that histone H3K9 trimethylation level was significantly upregulated compared with that of saline-treated groups (Figure 3B, *P* < 0.05).

### Increased GABA-Aα5 Histone H3K9 Acetylation Could Be Inherited by the Offsprings

**FIGURE 4:**
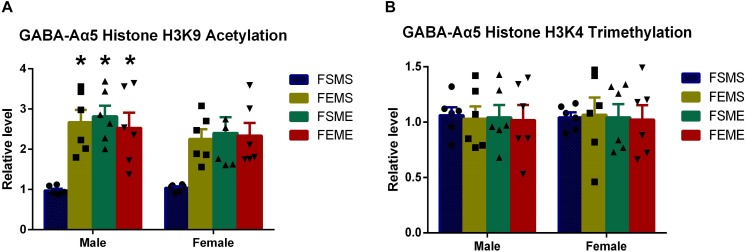
GABA-Aα5 histone H3K9 acetylation level was upregulated in the offsprings with an ethanol genetic background **(A)**, however, no significant difference showed in histone H3K9 trimethylation in the offsprings **(B)**. MSFS, male saline × female saline; MEFE, male ethanol × female ethanol; MSFE, male saline × female ethanol; MEFS, male ethanol × female saline. All data are presented as mean ± SD (*n* = 6). ^∗^*P* < 0.05 compared with control group.

It is generally acknowledged that partial epigenetic modifications can be inherited by the offsprings (Korpi et al., 2015). For further elucidation, we used the saline/ethanol-treated rats for breeding to randomly generate four groups of the offsprings (details refer to section “Animal Experiments”). At 2 months postnatal age, we sacrificed the offsprings for ChIP in the hippocampus. Results showed that the level of histone H3K9 acetylation in MSFE, MEFS, and MEFE groups were significantly higher than that of MSFS (Figure 4A, *P* < 0.05). However, no significant difference was found on histone H3K9 trimethylation level (Figure 4B).

### GABA-Aα5 Expression Was Elevated in the Offsprings With an Ethanol Genetic Background at the mRNA Level

**FIGURE 5:**
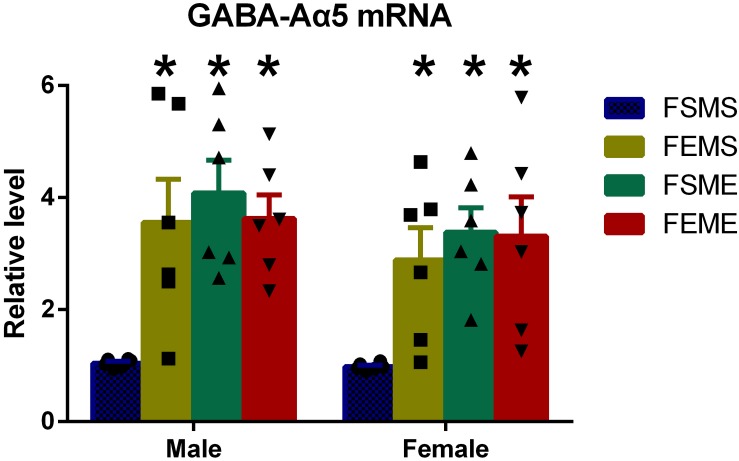
GABA-Aα5 expression was elevated in the offsprings with an ethanol genetic background at mRNA level. All data are presented as mean ± SD (*n* = 6). ^∗^*P* < 0.05 compared with the control group.

One step further to investigate whether the increased histone H3K9 acetylation had affected GABA-Aα5 expression, we detected GABA-Aα5 expression at the mRNA level by PCR. Results showed that in the offsprings with genetic background (MSFE, MEFS, and MEFE), all the mRNA levels of GABA-Aα5 expressions were significantly higher than those of MEFE (Figure 5, *P* < 0.05).

### Spatial Learning and Memory Deficits Were Detected in the Offsprings With an Ethanol Genetic Background

**FIGURE 6:**
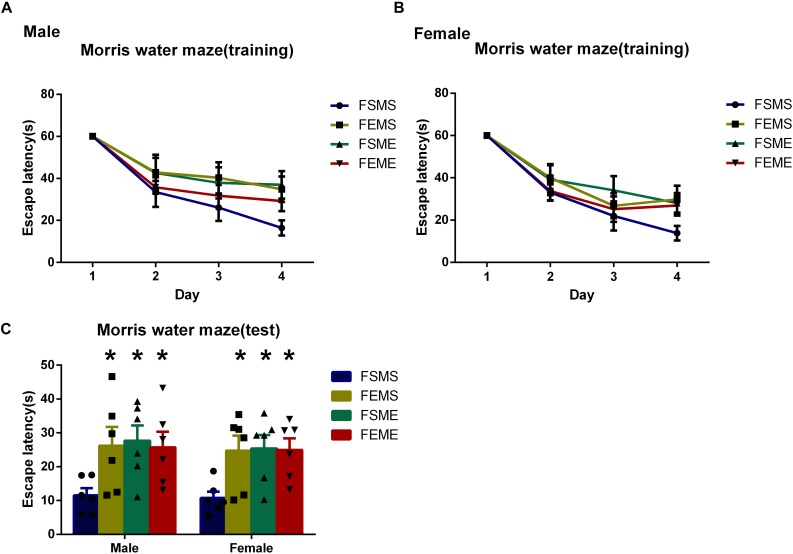
Spatial learning and memory deficits were detected in the offsprings with an ethanol genetic background in Morris water maze tests (MWMTs). **(A)** Escape latency of male offsprings training period. **(B)** Escape latency of female offsprings training period. **(C)** Escape latency of both male and female offsprings in the test. All data are presented as mean ± SD (*n* = 6). ^∗^*P* < 0.05 compared with the control group.

To detect the spatial learning and memory ability, MWM tests were conducted among all the offsprings. In the first day, there was no significant difference in escape latency among the four offspring groups. However, in the fifth day, both male and female offsprings with genetic background spent longer time to reach the platform than did MSFS (Figure 6, *P* < 0.05). Meanwhile, similar to MWM results of parental rats, the probabilities of MSFE, MEFS, and MEFE to reach the platform were significantly lower than those of MSFS during the trial.

## Conclusion and Discussion

Despite tremendous studies focusing on GABA receptors and alcohol-induced learning-memory dysfunction, the mechanism and whether the impairment will be inherited by the next generation remained unclear. This work was designed to investigate these questions. Our results showed that the upregulated GABA-Aα5 expression in the hippocampus might be involved in the spatial learning and memory deficits induced by chronic ethanol treatment. Furthermore, the increased level of GABA-Aα5 expression was demonstrated to be induced by GABA-Aα5 histone H3K9 acetylation, which directly led to GABA-Aα5 mRNA upregulation.

Epigenetic regulation, including histone modifications, DNA methylation, and non-coding RNAs, can mediate long-lasting changes in gene expression, which makes it a possible mechanism for the stable behavioral inheritance of the next generations (Nestler, 2014; Nagy and Turecki, 2015). In this paper, we found that the histone H3K9 acetylation could be inherited by the offsprings, which, as a result, induced GABA-Aα5 mRNA and GABA-Aα5 expression upregulation. Meanwhile, the level of histone H3K9 trimethylation was also increased after chronic ethanol treatment, however, as our results showed, it could not be inherited by the offsprings. Noteworthy, it is also suggested that the offsprings were vulnerable to suffer from spatial learning and memory deficits. Based on aforementioned, it is proposed that GABA-Aα5 histone H3K9 acetylation might participate in learning-memory dysfunction in the offsprings. All in all, our results may partially explain the mechanism that neuroimpairment induced by ethanol could be inherited via GABA-Aα5 histone H3K9 acetylation, which directly triggered the increase of GABA-Aα5 expression. Herein, GABA-Aα5 inhibitors could serve as leads for therapeutics.

However, this study still has some concerns to solve. According to the statistics from the WHO, more than three quarters of alcohol-induced deaths were among men (World Health Organization [WHO], 2018) however, our results did not show any significant differences between male and female gender, which still needs to be further explored. Besides, to the best of our knowledge, no consensus has been reached on connections between ethanol treatment and GABA-Aα5 expression. Centanni et al. (2014) proved that chronic intermittent ethanol treatment during adolescence (5 g/kg, 35% ethanol) for 48 h reduced GABA-Aα5 mRNA levels, whereas it has no effect on GABA-Aα5 expression. In contrast, none of above effects were observed in treatment during adulthood. Mahmoudi et al. (1997) found that 60 repeated intoxicating doses (5 g/kg, 25% ethanol) and repeated withdrawal episodes had no influence on GABA-Aα5 expression. However, Charlton et al. (1997) suggested that after 12 weeks of 5% ethanol consumption, the GABA-Aα5 expression increased in the hippocampus, while 4 weeks of ethanol treatment did not alter the GABA-Aα5 expression. These researches indicated that different doses, administration routes, and processing time of ethanol could possibly cause various GABA-Aα5 expression responses in the hippocampus. Hence, one step further to systematically evaluate the effect of ethanol treatment on GABA-Aα5 in various conditions was needed.

## Data Availability Statement

All datasets generated for this study are included in the manuscript/supplementary files.

## Ethics Statement

This study is approved by Wuhan Mental Health Center Ethics Committee at October 17, 2017.

## Author Contributions

XL and WH designed the study and provided most of the funds. KZ, XD, JJ, and AX supervised the study and wrote the manuscript. AX and MJ analyzed the data.

## Conflict of Interest

The authors declare that the research was conducted in the absence of any commercial or financial relationships that could be construed as a potential conflict of interest.
